# Effectiveness of family doctor contract services for chronic diseases management in China: a mixed-methods systematic review

**DOI:** 10.1186/s12875-025-03009-3

**Published:** 2025-10-31

**Authors:** Zhaochen Jiang, Dihan Tang, Mingsheng Chen, Lei Si

**Affiliations:** 1 School of Health Policy and Management, Nanjing Medical University, 101 Longmian Avenue, Nanjing, Jiangsu China; 2 School of Health Sciences, Western Sydney University, Campbelltown, NSW Australia; 3 NICM Health Research Institute, Western Sydney University, Westmead, NSW Australia

**Keywords:** Family practice, Chronic disease, Primary health care, Health services accessibility

## Abstract

**Background:**

The world is facing increasing challenges related to the rising burden of chronic diseases as the population ages. The family doctor contract service (FDCS) has been shown to play a vital role in managing chronic diseases in China. However, a systematic evaluation of its effectiveness is lacking. This systematic review explores the effectiveness of the FDCS in the management of chronic diseases and discusses the barriers and facilitators that affect their effectiveness in China.

**Methods:**

PubMed, Embase, Web of Science, CNKI and WanFang were searched for relevant studies, which were synthesised following their review using mixed methods. Articles on the effectiveness, barriers, and facilitators of FDCS that were published before 13 September 2024 were retrieved. We excluded studies that did not report the effectiveness of FDCS in managing chronic diseases and those that reported the barriers and facilitators of FDCS contraction. We report our review in accordance with the Preferred Reporting Items for Systematic Reviews and Meta-Analyses guidelines.

**Findings:**

We identified 7486 titles, retrieved 315 full-text articles and included 64 articles in the final analysis. The review found that 77% (*n* = 49) of the included studies reported positive effects of FDCS on blood pressure control, glucose control, health knowledge, and quality of life (QoL). The findings of the studies on medical expenses (*n* = 6) and quality of primary care (*n* = 4) were inconsistent. The following factors associated with the effectiveness of FDCS were identified from four qualitative and three mixed-methods studies; policy support, team building, and the needs of the residents. However, these studies also highlighted the need for improvements in FDCS related areas, including inadequate human resources and professional capabilities, imperfect economic incentive mechanisms, non-standard service implementation and management, low public participation and awareness, and inadequate technical support and resource guarantee. While 71.9% of the quantitative studies had moderate risks of bias, their quality was unsatisfactory.

**Conclusions:**

FDCS is effective for managing chronic disease. However, long-term effects have not been reported in previous studies. Further studies are required to identify the service cascade and maximise the effectiveness of FDCS. Efforts should also be made to address the challenges associated with FDCS in human resources, incentive systems, resource integration, and policy promotion to facilitate sustainable development in China.

**Registration:**

The review was registered in PROSPERO (registration no. CRD42024587272).

**Supplementary Information:**

The online version contains supplementary material available at 10.1186/s12875-025-03009-3.

## Introduction

With its ageing population, China faces the challenges of an increase in the prevalence and burden of chronic diseases, with 90% of deaths attributable to chronic diseases [1]. The primary health care system plays an important role in the management of chronic disease and health promotion [2]. Family doctors play important roles with support from funding from various sources. For example, the National Health Services in the United Kingdom is characterised by universal coverage, on-demand service, state-funded financing and free access for all [3]. In the United States, the family doctor system originated in the 1960 s and integrates health management into community general practice services to provide active health management for patients with chronic diseases [4, 5].

The development of primary health care in China started relatively late. China faced new challenges after two phases of equity-centred and efficiency-oriented primary health care, and initiated a new round of reforms in 2009. The outbreak of severe acute respiratory syndrome in 2003 highlighted the negligence of the health sector and exposed the weaknesses of the public health system [6]. The period from 2004 to date marks a new era of medical reform, during which the government has sought to balance fairness and efficiency in the development and rebuilding of the primary health care system. China initiated a new round of healthcare reform in 2009 to enhance primary medical and health services and enable general practitioners to provide health services in health facilities and at home. These general practitioners are also referred to as family doctors [7]. China began implementing the National Essential Public Health Services in the same year. These services, which are funded by the Chinese government, aim to provide affordable, equitable, high-quality, and accessible services [8]. The programme encompasses 14 key services, and hypertension and diabetes are the priority conditions for chronic disease management [9]. Several policy papers were published to promote family doctors as ‘health gatekeepers’ funded by the family doctor contract service (FDCS) [10, 11]. FDCS refers to a model in which residents enter into a service agreement with the service team of a community medical institution led by general practitioners. This agreement establishes a long-term and stable contractual relationship under which the team provides continuous, comprehensive, and personalised health management services to the participating residents [12]. Hypertension and diabetes are key chronic disease of concern during the implementation of FDCS, which is closely Linked to the National Essential Public Health Services. The National Health Commission has stipulated that the coverage rate of FDCS will exceed 75% by 2035. The coverage rate of FDCS for key groups is expected to exceed 85%, and the satisfaction rate is expected to be approximately 85% by the same year [13].

The number of primary health care providers increased 15-fold from 64,120 in 2008 to 1,016,238 in 2023 [14, 15], while the number of family doctors per 10,000 people increased from 1.07 in 2013 to 3.99 in 2023 [15, 16]. Approximately 70% of Chinese patients have access to healthcare services to manage chronic diseases [17], but the outcome of disease management is suboptimal [18]. For example, one study reported that nearly half of Chinese people aged 35–75 years have hypertension; however, fewer than one-third are being actively treated, and fewer than one in 12 have adequate blood pressure control [19]. Additionally, a 2019 survey showed that the use rate of FDCS in China was only 6.9% [20]. At present, findings on the effectiveness of FDCS in chronic disease management are varied. Some studies suggest that FDCS can effectively enhance disease cognition and self-management abilities of patients, leading to stable condition control through regular follow-up, personalised guidance and other interventions [21, 22]. However, other studies indicate that factors such as personnel shortages, limited service coverage, and low resident participation hinder the effectiveness of FDCS [23, 24]. In addition, most existing research focuses on a single dimension or specific region and lack systematic integration and comprehensive analysis, which limits its applicability to broader decision-making and practice. Therefore, this study adopts a systematic review approach to comprehensively summarise the effectiveness of FDCS in chronic disease management, identify the barriers and facilitators impacting its effectiveness, and provide a scientific foundation for optimising services and enhancing chronic disease management.

## Methods

### Data sources and search strategy

This study follows a convergent mixed-methods design framework, where both qualitative and quantitative data are collected simultaneously and then synthesized to derive more comprehensive conclusions. The review was registered in PROSPERO (registration no. CRD42024587272). We used EndNote X9 (Clarivate, London, UK) to manage the retrieved literature, and we adhered to the Preferred Reporting Items for Systematic Reviews and Meta-Analyses 2020 checklist to ensure reporting quality [25].

PubMed, Embase, Web of Science, CNKI, and WanFang were searched for studies published from database inception to September 13, 2024. The search strategy used for PubMed included the following search terms: (‘family doctor*’) OR (‘family physician*’) OR (‘general practi*’) OR (general practice [MeSH]) AND China AND (chronic disease*) OR (chronic care) OR (diabetes) OR (hypertension) OR (Chronic Disease[MeSH]) OR (Long-Term Care[MeSH]) AND (English[Language]) OR (Chinese[Language]). These terms were adapted for searching the other databases. Additional details of the search syntax are provided in the Supplementary File (Table S1).

### Inclusion and exclusion criteria

Studies were included if they (1) published in a peer-reviewed journal; (2) written in English or Chinese; (3) discussed an intervention by a family doctor contracting service in China; (4) reported outcomes of chronic diseases management, such as hypertension control rate, systolic and diastolic blood pressures, diabetes control rate, fasting blood glucose concentration, and quality of life; and (5) reported facilitators and barriers to the effectiveness of FDCS. Conversely, studies that (1) were reviews, comments, book chapters, grey literature, conference papers, abstracts, reports, dissertations or protocols; (2) had unavailable full texts; (3) were written in languages other than English or Chinese; (4) had no comparison or involved an unspecific comparison; or (5) identified the facilitators and barriers to contracting FDCS were excluded from this review.

### Screening process

The results from the database were exported to EndNote, and duplicate articles were removed. Two researchers (Z.J. and D.T.) independently screened the titles and abstracts of the retrieved studies based on the inclusion and exclusion criteria. The full texts of the studies deemed relevant (including those with disagreements between the two reviewers) were extracted and further scrutinised by the same reviewers. Discrepancies were resolved through discussions involving a third reviewer (L.S.).

### Data extraction

We used Excel 2016 for data extraction. The data were extracted from the final selection of articles by the two aforementioned researchers (Z.J. and D.T.) using a form to capture key information. The date extracted from the quantitative studies included the year of publication, study design, location, participants, sample recruitment, research purpose and main findings. The date extracted from the qualitative studies included the data collection methods, stakeholders interviewed in the included qualitative studies, and identified facilitators and barriers. The date extracted included the quantitative and qualitative aspects of the mixed-methods studies. The data were cross-checked, and any discrepancies were resolved by consensus.

### Quality assessment

We adopted the following three instruments to evaluate the quality of the reports of included studies based on the study design. The 10–13-item Joanna Briggs Institute Critical Appraisal Tools checklist was adopted for quantitative studies [26] such as case–control studies, cohort studies and randomised controlled trials. The 10-item Critical Appraisals Skills Programme was used to evaluate the quality of reports of qualitative studies [27]. The 17-item Mixed-methods Appraisal Tool was used to evaluate the quality of reports of mixed-methods studies [28]. Two researchers (Z.J. and D.T.) independently appraised the quality of the included articles and reviewed them together until a consensus was reached. They categorised the studies into high-risk (0–3 points for quantitative studies; 0–3 points for qualitative studies; 0–5 points for mixed-method studies), moderate-risk (4–6 points for quantitative studies; 4–8 points for qualitative studies; 6–12 points for mixed-method studies), and low-risk (7–13 points for quantitative studies; 9–10 points for qualitative studies; 13–17 points for mixed-method studies) groups [29]. Only the studies with low or moderate risks of bias were included in the final data syntheses.

### Data synthesis

We elected to employ narrative synthesis without meta-analysis as our analytical approach because of the heterogeneity of the research themes in the included studies. We identified and collected all the themes from the studies based on the results of extracted data. The data were classified based on the similarity of their meanings and assigned to different themes. The categories with similar meanings were synthesised and integrated to derive comprehensive findings.

## Results

### Characteristics of the included studies

**Fig. 1:**
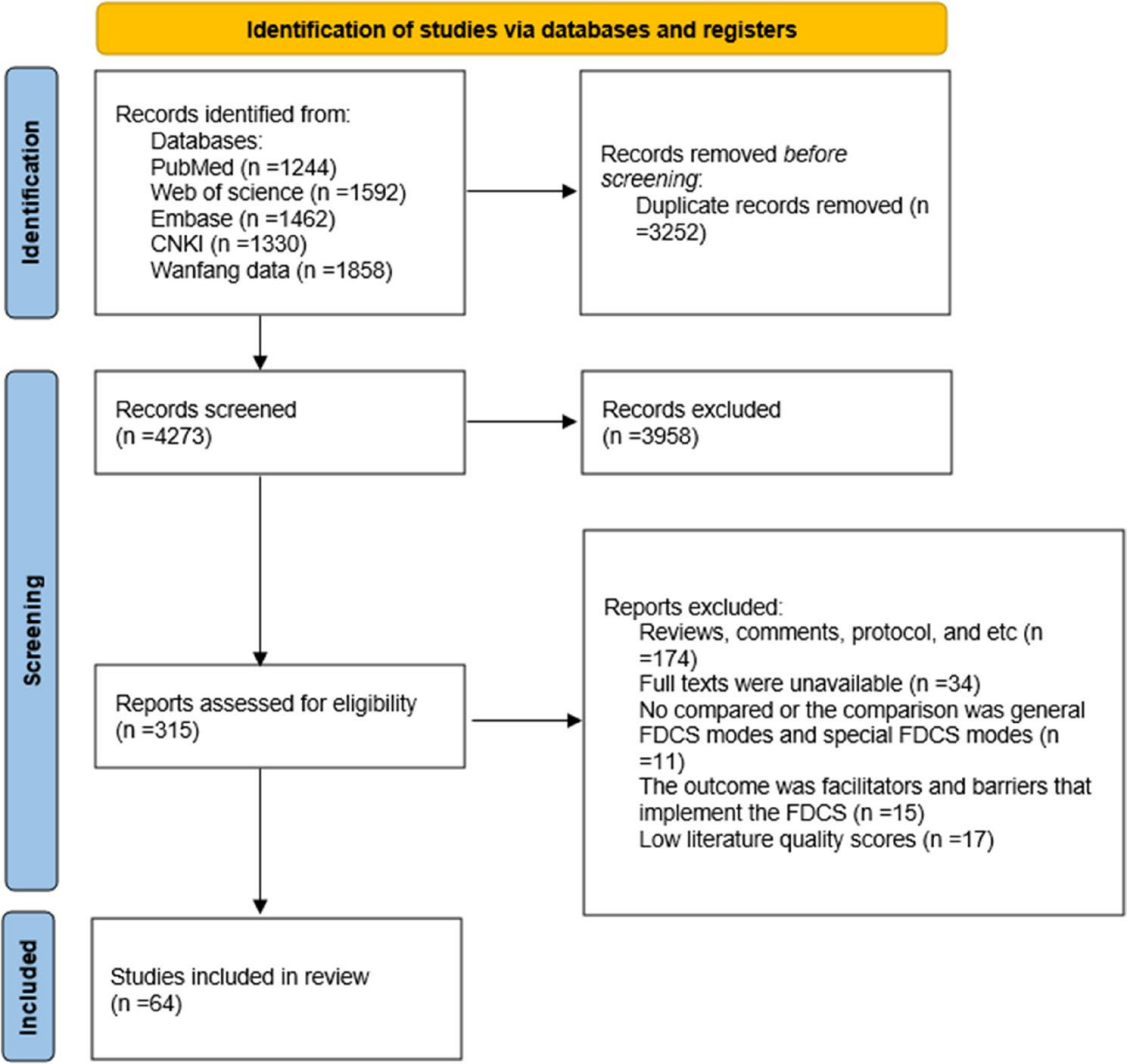
PRISMA flow diagram

The search of the five databases yielded 7486 studies. The titles and abstracts of 4273 articles were screened after removing duplicates, and 315 articles were subsequently selected for full-text review. Finally, 64 studies were included in the review. The study characteristics are summarised in Tables 1, 2 and 3. Of the 64 studies, 57 (89.1%), 4 (6.2%), and 3 (4.7%) had quantitative, qualitative, and mixed-methods designs, respectively. The screening process is shown in Fig. 1. Eleven and 46 of the quantitative studies were cohort and cross-sectional studies, respectively. The follow-up durations for 5 cohort studies were less than 3 years. All the studies included in the analysis were published after 2013, and 2022 had the highest number of studies (*n* = 12, 18.7%). Thirty-four (53.1%) studies were conducted in eastern China. Additional details of the publication trends and regional distributions are provided in the Supplementary File (Figures S1 and S2).

**Table 1:** Characteristics of included qualitative studies

Reference	Location and time	Participants	Design	Facilitators and barriers	Quality of study
Yuan S, et al., 2019 [23]	Zhejiang, Jiangsu, Fujian, Shanxi, Hubei, Anhui, Qinghai, Sichuan and Chongqing 2017	62 policy makers; 19 leaders; and 48 family doctor team members	Focus group discussions Individual interviews	Facilitators: National reform involving both top-down and bottom-up policy making; support from essential public health funds, fiscal subsidies and health insurance; extra performance-based payments for family doctor teams based on evaluation; and positive engagement of health administrators. Barriers: A lack of essential matching mechanisms at national level; distrust in the quality of primary care, lack of government subsidies and health insurance reimbursement and performance ceiling policy; low competency of family doctors and weak influence of evaluations on performance–based salary; and misunderstanding about family doctor contracting services.	10
Zhou H, et al., 2015 [89]	Jiangxi 2013	8 managers, 20 village doctors, and 11 villagers	Semi-structured interviews	Facilitators: Increase in public health services to the villagers; patient satisfaction with the doctor–patient relationship. Barriers: Heavy workload, insufficient remuneration, staff shortage, lack of official identity and ineffective performance appraisal	10
Zhou H, et al., 2015 [24]	-	84 health providers and 20 demanders	Document collection, non-participant observation and interviews	Barriers: Insufficient incentives	9
Zheng J,2023 [88]	Anhui 2020	11 contracted residents	Interview	Facilitators: Variety of contracted service models Barriers: Insufficient publicity	9

**Table 2:** Characteristics of included mixed-methods studies

Reference	Location and time	Qualitative participants	Participants and sample recruitment	Design	Facilitators and barriers	Main findings	Quality of study
Liu S, et al., 2022 [87]	Shandong 2020	15 family doctors and 11 contracted residents	1098 Stratified random sampling	Interview Cross-sectional design	Barriers: Low enthusiasm, referral work, inadequate special medical services	The score of Continuous dimension is relatively high and the score of Coordinated dimension is relatively low; Among the three derived dimensions, the score of Family-centredness dimension is relatively high, and the score of Culturally-competent dimension is low.	17
Zhang Z, Zhang R, Peng Y, et al., 2023 [59]	Beijing 2020–2021	30 family doctors	283 Cluster sampling	Interview Cross-sectional design	Facilitators: Doctor-patient trust relationship, humanistic care services Barriers: High risks in the process of home visits, a lack of supervisory and incentive mechanisms, insufficiency of time and energy	Primary care is the most common services for disabled older adults, followed by health consultation and education, and medication examination. Home visits are among the most desired services for disabled older adults, which is a bridge of effective communication between family doctors and disabled older adults. And family doctors, disabled older adults and their families have a good cooperation	17
Li H, 2021 [35]	Henan 2020–2021	12 family doctors	500 Simple random sampling	Interview	Barriers: Communication between high- and low-level hospitals needs to be strengthened	Better blood pressure and blood sugar control for residents signing up with family doctors	12

**Table 3:** Characteristics of included quantitative studies

Reference	Location and time	Participants and sample recruitment	Design	Main findings	Quality of study
Han D, Guo X, 2022 [63]	2020	82	Observational study	Contracted residents scored higher than uncontracted residents in dietary knowledge, medication knowledge, exercise knowledge and pathology knowledge, while also demonstrating greater management effectiveness and better adherence.	4
Zhu G, et al., 2020 [78]	—	379 Simple random sampling	Observational study	Residents reduced cost of treatment and hospitalization after contracting	6
Sun G, 2021 [30]	2019–2021	96	Observational study	Health knowledge, self-behavioural control, and blood pressure and glucose control of contracted residents are better than those of uncontracted residents	4
Mo H, et al., 2017 [38]	2014–2015	88	Observational study	Contracted residents had better lipid biochemical indexes, blood pressure control effect, blood glucose control effect, and survival quality score effect	5
Yin H, 2018 [64]	2016 − 1017	126	Observational study	Contracted residents had higher health literacy scores and lower scores for adverse moods of anxiety and depression than uncontracted residents	5
Liu H, Liu D, 2023 [6]	Sichuan 2023	1600 Multi-stage sampling	Observational study	The total health literacy score, health knowledge and concepts, health behaviours, and diabetes prevention and treatment outcomes of contracted residents were better than those of uncontracted residents	9
Tan H, Wei L, 2020 [66]	2018–2019	120	Observational study	Contracted residents had higher knowledge acquisition scores	5
Li H, 2019 [41]		240	Observational study	Contracted residents had higher health literacy scores and better blood pressure and glucose control	4
Zhao H, 2019 [67]	Jiangsu 2016-2019	100	Observational study	Contracted residents achieved a higher effective rate in the control of blood glucose, blood pressure and lipids compared with uncontracted residents, while contracted residents also showed a higher score in knowledge mastery and a lower percentage of risky behaviours than uncontracted residents	4
Wang J, 2019 [68]	Shanghai 2016-2018	100	Observational study	Contracted residents had higher chronic disease treatment effectiveness and health awareness than uncontracted residents	4
Feng J, 2023 [52]	Beijing 2020-2021	80	Observational study	Contracted residents had better blood pressure control and health knowledge scores than uncontracted residents	4
Wang J, et al., 2019 [60]	Guangdong 2016-2018	400	Observational study	Contracted residents had better chronic disease treatment efficiency and follow-up consultations than uncontracted residents	4
Wang J, et al., 2014 [31]	Shanghai	300 Multi-stage sampling	Observational study	Health behaviours of contracted residents improved, but blood pressure and blood glucose control rates varied at both baseline and at the time of the outcome survey	4
Huang J, et al., 2018 [75]	Shanghai 2013, 2016	3040 Multi-stage sampling	Observational study	Personal cash expenditures of contracted residents are higher than those of non-contracted residents; family physicians’ control over personal cash expenditures increases over time	4
Chen J, 2018 [61]	2016–2018	200	Observational study	Contracted residents had fewer risk factors than uncontracted residents and had better scores for health knowledge, self-control, and adherence than the uncontracted	4
Chen J, et al., 2013 [69]	Shanghai 2012	300 Simple random sampling	Observational study	Contracted residents had more knowledge related to blood pressure	4
Wei L, et al., 2018 [47]	Anhui 2016-2017	300	Observational study	Contracted residents had better control of hypertension and better control of health behaviours	4
Deng L, et al., 2016 [42]	Chongqing	281	Observational study	The biochemical indicators of blood glucose, blood pressure and blood lipids of contracted residents are better than uncontracted residents	4
Song L, 2021 [71]	Jiangsu 2018-2019	98	Observational study	Contracted residents had better self-control, health knowledge and adherence than uncontracted residents	4
Wu L, et al., 2020 [36]	Anhui 2014-2019	4488	Observational study	The rate of blood glucose and blood pressure control among contraction residents was higher than uncontracted residents	4
Feng Q, et al., 2020 [72]	Shanghai 2018-2019	60	Observational study	Contracted residents had better health knowledge than the control group and lower maladaptive behaviours than uncontracted residents	4
Gu Q, 2017 [43]	Zhejiang 2016-2017	110 Simple random sampling	Observational study	Contracted resident knowledge rate, blood glucose and blood pressure indicators, blood glucose normalisation rate and effective rate, and blood pressure normalisation rate were better than uncontracted residents; there was no difference between the two groups in blood pressure effective rate	4
Deng S, 2015 [44]	2013–2015	400 Simple random sampling	Observational study	Contracted residents had better knowledge, self-control, blood pressure and blood glucose biochemical indexes, effective rate, and normal rate than uncontracted residents	5
Chan W, et al., 2023 [81]	Guangdong 2022-2023	96	Observational study	Contracted residents had better adherence and higher quality of life scores	4
Lu W, et al., 2016 [62]	Shanghai	2886 Multi-stage sampling	Observational study	Contracted residents had better self-management and disease awareness than uncontracted residents	5
Lin W, t al, 2020 [32]	Shanxi	558	Observational study	Contracted residents had better hypertension and diabetes control rates	4
Luo W, et al., 2017 [73]	Shenzhen 2015-2017	200	Observational study	Contracted residents had better health literacy scores, blood pressure, blood glucose, and lipid interventions	4
Zhu X, 2020 [57]	Guangdong 2017-2018	400	Observational study	Contracted residents had better fasting blood glucose, medication adherence, self-health management behaviours, and quality of life	4
Lu X, 2021 [53]	2019	100	Observational study	Contracted residents had lower blood pressure levels than uncontracted residents	4
Li X, 2020 [33]	2018–2019	208	Observational study	Contracted residents had a higher rate of medication adherence, blood glucose, blood pressure, blood lipids, and body fat normalisation than uncontracted residents	4
Chen Y, 2020 [82]	Jiangxi 2017-2019	80	Observational study	Contracted residents had higher treatment adherence and quality of life scores o than uncontracted residents	4
Deng Y, et al., 2022 [49]	Beijing 2020-2021	102	Observational study	Contracted residents had lower diastolic blood pressure, systolic blood pressure, glycosylated haemoglobin, postprandial blood glucose, and fasting blood glucose than uncontracted residents, and had better self-control	4
Zhang Y, et al., 2023 [34]	Shanghai 2019-2020	878 Multi-stage sampling	Observational study	Contracted residents had higher rates of blood pressure, blood glucose, blood lipids, and weight compliance than uncontracted residents	8
Jing Y, et al., 2013 [50]	Sichuan	440 Stratified sampling	Observational study	Contracted resident health awareness and compliance increased, their blood pressure and blood glucose conditions improved, and their treatment costs decreased. However, there is still room for improvement in the quality and effectiveness of health management services	5
Hu Y, et al., 2019 [48]	Jiangsu 2018	300	Observational study	The systolic and diastolic blood pressure in contracted residents were significantly lower than that uncontracted residents after the intervention; the knowledge of hypertension and self-management ability of contracted residents were significantly higher.	4
Li Y, et al., 2022 [39]	Shandong 2019-2020	106	Observational study	Contracted residents had higher scores of health knowledge, self-control and adherence, various quality of life scores, blood pressure, blood lipids, and glucose standardisation rate, and satisfaction than uncontracted residents	4
Wang Y, 2022 [77]	China 2015, 2018	2950	Observational study	Family doctor contract services have significantly reduced the total cost of outpatient care and out-of-pocket expenses for older persons	6
Yun Z, 2021 [46]	2018–2019	64	Observational study	Contracted residents had better health knowledge scores, self-management ability scores, blood glucose, blood lipids and blood pressure than uncontracted residents	4
Yang Z, 2022 [55]	2021	102	Observational study	Contracted residents had greater blood pressure reduction, higher health literacy scores and better adherence	4
Zhou Z, 2018 [45]		100	Observational study	Contracted residents had better disease knowledge rate, blood pressure and blood glucose levels than uncontracted residents	4
Huang Z, et al., 2015 [37]	Shenzhen 2013-2014	234	Observational study	Contracted residents had better control rate of hypertension and diabetes mellitus than uncontracted residents had better, and the quality of life was higher than uncontracted residents had better	4
Shi Z, 2022 [40]	Beijing 2018-2021	68	Observational study	The health knowledge score of contracted residents was better than that of uncontracted residents, and contracted residents had a higher rate of blood pressure compliance, blood glucose compliance, and blood lipid compliance than uncontracted residents	5
Feng S, et al., 2020 [84]	Guangdong 2015	828 Multi-stage sampling	Observational study	Contracted patients reported higher scores in dimensions of PCAT total score, first contact-utilisation, first contact-accessibility, continuity, coordination (referral), comprehensiveness (utilisation), comprehensiveness (provision), family-centredness, community orientation than uncontracted.	8
Huang J, et al., 2019 [74]	Shanghai 2014	2886	Observational study	Doctor-visiting behaviour: Contracted residents more frequently visited their doctors. The referral rate for contracted residents was significantly higher too. Community health management for chronic diseases: The contracted residents had a higher rate of self-management than uncontracted residents Personal health spending for medical care: The contracted residents were found to spend significantly more than uncontracted residents Satisfaction: Contracted residents were significantly more satisfied with the care they received	8
Huang J, et al., 2019 [21]	Shanghai 2013,2016	1734 Multi-stage cluster sampling	Observational study	More contracted residents engaged in self-management under the guidance of family doctors than uncontracted residents Effectiveness of self-management: Contracted residents had more effective self-management	8
Lai S, et al., 2021 [22]	Shaanxi 2018	6503 Multi-stage, stratified cluster random sampling	Observational study	Contracted residents had significantly higher quality of life than uncontracted residents. The inequity in quality of life among contracted residents was significantly lower than that among uncontracted residents	8
Li L, He X and Zhang C, 2022 [76]	Beijing 2018–2021	5851	Observational study	Family doctor contract services significantly increased the frequency of resident medical visits to community hospitals; Family doctor contract services significantly increased resident medical expenses.	9
Li L, Zhong C, Mei J, et al., 2018 [85]	Guangzhou 2014	692 Multi-stage sampling	Observational study	The total PCAT score, continuity, comprehensiveness and family-centredness were higher for contracted than for the uncontracted. The score of first-contact utilisation coordination were lower for contracted than for the uncontracted.	8
Li Z, et al., 2021 [79]	Shandong 2018	1210 Multi-stage random sampling	Observational study	The association between family doctor contract services and quality of life among patients with chronic disease is significant. This relationship varied with income levels and educational attainment.	8
Liao J, et al., 2021 [86]	Guangdong 2019	1185 Three-stage cluster sampling	Observational study	Family doctor contract services play a unique role in improving the quality of primary care experienced by patients with several chronic diseases.	8
Lin W, et al., 2023 [58]	Shenzhen 2020	4389 Multi-stage sampling	Observational study	Women who contracted had more HPV-related knowledge, participated more in screening, and greater willingness to engage in future screening than the uncontracted.	8
Liu T, et al., 2019 [51]		471	Observational study	Family doctor contract services can effectively improve blood pressure control of patients with hypertension and improve their quality of life.	9
Wang L and Liu W, 2020 [80]	CHARLS	382	Observational study	Family doctor contract services probably had an influence on the quality of life by significantly improving the role-emotional. However, the improvement of the other seven dimensions of quality of life was not significant.	9
Xu C, et al., 2022 [56]	Zhejiang 2017–2019	2310	Observational study	Family doctor contract services can improve glycaemic and lipid control and reduce the incidence of related complications with diabetes	8
Chen Y, et al., 2022 [54]	Shandong 2018	982 Multi-stage stratified random sampling	Observational study	Compared with uncontracted patients, contracted patients reported greater hypertension management awareness.	8
Han Y, 2021 [83]	Jiangsu 2018-2020	80	Observational study	Quality of life scores were higher in the test group than in the control group	4
Feng L, et al., 2019 [70]	Shandong 2018	204	Observational study	Contracted residents had significantly higher scores of health knowledge than uncontracted residents	4

Note: In the table, PCAT stands for primary care assessment tool. And HPV stands for human papilloma virus

All 64 included studies were focused on chronic disease domains, including hypertension [30–55], diabetes [30–46, 49, 50, 56, 57], lipid abnormalities [33, 34, 40, 46], other chronic diseases [58–60], health knowledge [21, 30–32, 40–45, 47, 48, 57, 61–73], health costs [62, 74–78], quality of life (QoL) [22, 38, 39, 51, 57, 61, 79–83], quality of primary care [84–86], and barriers and facilitators to the effectiveness of FDCS [23, 24, 35, 59, 87–89]. We divided these dimensions into three, health status and health outcomes, health costs and quality of primary care, and facilitators and barriers. The included studies demonstrated that FDCS had a positive effect on health outcomes and quality of primary care, but there was no definite report on health costs. Of the studies, 25 (39.1%) reported multiple quantitative outcomes, as shown in Table 4.

**Table 4:** Summary of studies that reported multiple outcome measures

Study	Hypertension	Diabetes	Lipid abnormalities	Health knowledge	Quality of life	Healthcare costs
Chen J, 2018 [61]				✓	✓	
Lu W, et al, 2016[62]				✓		✓
Zhu X, 2020[57]		✓		✓	✓	
Sun G, 2021[30]	✓	✓		✓		
Li H, 2019[41]	✓	✓		✓		
Wang J, et al, 2014[31]	✓	✓		✓		
Deng L, et al, 2016[42]	✓	✓		✓		
Gu Q, 2017[43]	✓	✓		✓		
Deng S, 2015[44]	✓	✓		✓		
Lin W, et al, 2020[32]	✓	✓		✓		
Zhou Z, 2018[45]	✓	✓		✓		
Zhang Y, et al, 2023[34]	✓	✓	✓			
Wei L, et al, 2018[47]	✓			✓		
Hu Y, et al, 2019[48]	✓			✓		
Liu, T, et al, 2019[51]	✓				✓	
Li H, 2021[35]	✓	✓				
Wu L, et al, 2020[36]	✓	✓				
Deng Y, et al, 2022[49]	✓	✓				
Jing Y, et al, 2013[50]	✓	✓				
Huang Z, et al, 2015[37]	✓	✓				
Mo H, et al, 2017[38]	✓	✓			✓	
Li X, 2020[33]	✓	✓	✓			
Li Y, et al, 2022[39]	✓	✓			✓	
Shi Z, 2022[40]	✓	✓	✓	✓		
Yun Z, 2021[46]	✓	✓	✓	✓		

### FDCS effects on health status and health outcomes

The impact of FDCS on health status and health outcomes includes its impact on hypertension, diabetes, lipid concentrations, and some other chronic diseases, as well as on QoL and health knowledge. Fifty-two (81.3%) of 64 included studies reported the effects of FDCS on health status and health outcomes. The three most frequent themes were health knowledge (*n* = 27), hypertension (*n* = 26), and diabetes (*n* = 21).

All 52 studies reported positive outcomes on the health status and health outcomes. Among the studies involved health knowledge, 23 studies were conducted on patients with chronic diseases [21, 30, 32, 40–45, 47, 48, 57, 61, 63, 64, 66–68, 70–73], others were conducted on residents or poor elderly individuals [31, 62, 65, 69]. Among the studies involved hypertension, 14 studies used systolic and diastolic blood pressures as evaluation indicators [41–53, 55], 11 studies used the hypertension control rate to evaluate the effectiveness of hypertension treatment [30–40], and one assessed blood pressure measurement awareness through questionnaires [54]. Among the studies involved diabetes, 11 studies used the diabetes control rate to evaluate the role of family doctors in the care of patients with diabetes mellitus [30–40], 10 studies used fasting blood glucose or 2-h postprandial glucose as evaluation indicators [41–46, 49, 50, 57]. Among the 11 studies involved QoL [22, 38, 39, 51, 57, 61, 79–83], five instruments were used in them, including the Short Form 36 (SF-36), EQ-5D-3 L, Generic Quality of Life Inventory 74, QoL-BREF and Diabetes Specific Quality of Life Scale. Among the studies involved lipid levels, three studies reported lipid control rates [33, 34, 40] and one study measured four biochemical indicators related to blood lipids [46]. Detailed subgroup results were in the Supplementary File (Tables S2-S9).

### FDCS effects on health costs and quality of primary care

Nine (14.1%) of the 64 included studies reported on health costs and quality of primary care. Six and three of these studies reported on health costs and quality of primary care, respectively [62, 74–78, 84–86].

Positive outcomes related to health costs and quality of primary care were reported by six studies (9.4%), Three studies used the Primary Care Assessment Tool (PCAT) and the Assessment Survey of Primary Care (ASPC) to assess the quality of primary care. The results showed that contracted residents scored higher. However, contradictory findings have been reported for first contact utilisation. Two study reported FDCS as a facilitator [84, 86], whereas another reported it as a barrier [85]. Two studies on health costs reported that FDCS facilitated the reduction of health costs. The results showed that the health costs of contracted residents were lower than that of uncontracted residents. The difference between the two groups was 846.8 yuan/year [78] and 468.93 yuan/year [77], respectively.

Among studies reported negative outcomes in health costs (*n* = 3, 4.7%), 2 studies reported the annual health costs of residents [62, 74], and the other reported the monthly health costs of residents at community hospitals [76].

One study reported ambivalent outcomes for health costs between the two rounds of the survey [75]. The study involved two surveys. The health costs of the contracted residents were higher based on the 2013 survey but lower based on 2016 survey [75].

### Facilitators and barriers to the effectiveness of FDCS

Seven (10.9%) of the 64 included studies reported on barriers and facilitators to the effectiveness of FDCS. Policy support, team building, and the needs of residents have been associated with positive outcomes in the implementation of FDCS [23, 35, 59, 88, 89]. The central government provided policy support by offering guidance for local implementation, summarising typical cases, and promoting pilot experiences [23]. Family doctor teams comprised multidisciplinary personnel, including general practitioners, nurses, and public health workers. It also received support from experts at senior hospitals, which enhanced the continuity and professionalism of services [35]. The aggravation of the ageing society and the increasing demand for chronic disease management have led to some residents receiving personalised health management through FDCS [23, 89].

However, FDCS also faces several barriers to its functioning [23, 24, 35, 59, 87–89].

#### Inadequate human resources and professional capabilities

The number of family doctors was insufficient, and the demand for high-quality of services was high. The doctors in some areas had a high workload [59, 88, 89], and their academic qualifications were generally low [23]. They often had limited expertise in general practice reasoning and chronic disease management, and relied heavily on experience-based medication use [35, 88].

#### Imperfect economic incentive mechanisms

The poor connection between basic public health funding and health insurance reimbursement policies has led to an imbalance between the income and workload of family doctors [23, 24, 59]. Additionally, the low performance pay and vague evaluation criteria have reduced the motivation of doctors [87, 89].

#### Non-standard service implementation and management

Non-standard service implementation and management have impeded the effectiveness of FDCS. The contracted services had fragmented characteristics, and the health records were incomplete [24, 89]. They also had a two-way referral mechanism that allowed patients to access higher-level hospitals without a referral order and reimbursement incentives for referrals [87].

#### Low public participation and awareness

Low health awareness among residents, their focus on treatment rather than prevention, and misunderstanding about contracted services due to inadequate policy publicity contribute to low service compliance [24, 35, 59, 87, 88].

#### Inadequate technical support and resource guarantee

The lack of interoperability between medical and public health systems data necessitates information re-enter by family doctors and hinders efficiency [23]. Shortages of psychological and Chinese medicine services also limit the development of FDCS [35, 87].

### Quality assessment

All qualitative studies had low risks of bias. Two of the three mixed-methods studies had low risks of bias and one had moderate risk. Sixteen of the quantitative studies had low risks of bias and 41 had moderate risks. The quality assessment scores of the included articles are provided in Tables 1, 2 and 3.

## Discussion

### Main findings

To the best of our knowledge, this is the first study to systematically synthesise the effectiveness, barriers and facilitators of FDCS in China. The FDCS is effective for controlling blood pressure, improving QoL, enhancing primary care quality, regulating blood glucose concentrations, and increasing health knowledge. National reforms, patient demand, and team building enable the FDCS to be effective. However, low doctor motivation, residents’ issues, low referral efficiency and lack of special services hinder its effectiveness.

The results of this review are consistent with those of previous studies that evaluated the effectiveness of FPCS [90, 91]. The benefits of FDCS have also been reported in a review of its effectiveness for controlling hypertension in China [90]. A cross-sectional study conducted in the Eastern Mediterranean reported improved screening for hypertension and diabetes following the involvement of family doctors. However, the study also reported reduced hypertension treatment outcomes among contracted residents [91]. This may be because family doctors are more inclined to use non-pharmacological therapies, such as lifestyle improvement and the reduction of diet-related risk factors. The results may differ if the impact of family doctors is evaluated over a longer period. The studies on healthcare costs included in this review also highlight the dependence of the effect of FDCS on healthcare expenditures for the population on time. The effect of FDCS on controlling personal cash expenditures appears to increase over time. However, this review included fewer cohort studies, and the participants were not followed up for long durations. Most of the included studies had follow-up durations of only 1–2 years. Future studies should explore the long-term effects of FDCS.

The research findings indicate that contracting with family doctors can enhance the health knowledge of residents. Insufficient health knowledge is a better predictor of poor health than age, income, employment status, educational level, or race [92]. The FDCS is important for reducing the waste of medical resources and improving the health of residents.

### Healthcare costs

The analysis of the included studies revealed differing views among researchers on the impact of FDCS on the healthcare costs of residents. This highlights the need for further research. The impact of health status and age on medical costs was reported in detail for three of six studies included in the review [62, 74, 75]. Some studies have reported that the gap was significant only among older adults [62, 74], whereas the other has reported that it was significant among younger and middle-aged [75]. All the studies reporting the number of visits have indicated that signing up with a family doctor increases the number of visits [76, 78]. The results of the comparisons at different times points reveal that the impact of FDCS on costs increases gradually [75]. The observed differences in the research results may be due to various reasons. The first is the signing time. The impact of the duration of signing on medical costs has been reported [75]. However, most of the studies included in this review did not investigate the timing of the singing of the residents with their family doctors. Second, the choices of the respondents also affect the results. The differences in medical expenses disappear are nullified after controlling for health conditions [62, 74, 75]. The choices of the respondents may explain the different results. Five of the six studies were conducted in the eastern region. The remaining study used data from the China Health and Retirement Longitudinal Study. Notably, research on the impact of FDCS on medical costs in the central and western regions are lacking.

### Quality of primary care

Studies on quality of primary care have also reported divergent findings. The residents who contracted family doctors consistently had significantly higher PCAT scores. However, the findings on first-contact service utilization were inconsistent: one study reported that contracting family doctor was a hindrance [85], while the other two reported that it was a facilitator [84, 86]. All three studies were cross-sectional surveys conducted in Guangdong Province. The study reporting a hindering effect was conducted in 2014 [85], while those reporting facilitating effects were conducted in 2015 [84] and 2019 [86]. These heterogeneous results reflect the complexity of FDCS implementation outcomes. It highlights the need for further research to explore the impact of family doctors on quality of primary care and identify effective pathways through which FDCS can enhance it.

### Comparison with global models

Some family doctor systems have matured. The “gatekeeper” role of family doctors in the National Health Service of the UK highlights the gap in the FDCS in China in terms of referral efficiency and the first-visit system [93]. The public-private hybrid financing model in Australia is flexible, but it exacerbates the accessibility gap [94]. In contrast, the FDCS in China, which relies on the public system to ensure public welfare, has insufficient incentives for doctors. The proposal of interprofessional collaboration provides a direct reference for China to solve problems such as weak specialised collaboration and single services [95].

There are also studies on the effectiveness of family doctors internationally. For example, cross-sectional studies conducted in the Eastern Mediterranean region have identified several factors that hinder the effectiveness of the FDCS [91]. These barriers are largely consistent with those discussed in this study. While the results of quantitative studies indicate that FDCS has been implemented effectively in China and these barriers having a limited impact on its effectiveness, international studies suggest that certain factors may significantly undermine the effectiveness of the FDCS in the future. For example, the decline in primary care utilisation among populations in the United States [96], lack of knowledge about family doctors among and preference of Saudi Arabian residents for emergency room visits [97], and the misconception among German residents that family doctors do not treat obstetric and gynaecologic patients [98] among others, have undoubtedly weakened the role played by family physicians in primary health care and the health care system.

Scholars have increasingly conducted more in-depth research on factors that affect the effectiveness of family doctors. A study in the United Kingdom examined the response of family physicians to changes in the stringency of targets under financial incentives, and the results demonstrated the outcome of a strategic initiative in which family doctors were faced with stricter targets for care [99]. A cross-sectional study in Lebanon analysed family medicine from a public perspective and showed that education level, location and health care system structure led to different perceptions of family medicine [100]. In the United States, one study showed that health system factors were most closely associated with several factors that impede paediatric referrals [101]. These studies point to new directions for subsequent evaluation studies of FDCS in China. The subsequent work of government staff and academics may focus on addressing or improving these issues.

Interestingly, we found that increasing ageing was a contributing factor to the FDCS in China [23, 59]. It also been reported as a major challenge for family doctors in Australia [102]. This may be because the family doctor system in China is in its infancy, whereas it is relatively well established in Australia.

None of the studies included in this review addressed frailty. However, existing research has shown that incorporating frailty interventions into chronic disease care can enhance patient resilience, reduce the progression of chronic diseases, and improve overall health outcomes [103]. The future implementation of family doctors in China should consider interventions for frailty in patients with chronic diseases.

### Innovative value, methodological and policy implications

To the best of our knowledge, this study is the first to evaluate the effects of FDCS systematically. Mixed-methods studies can provide a more comprehensive description of the effects of FDCS than qualitative or quantitative studies. The bio-psycho-social model posits that the psychological makeup, physiology, spirit, body and internal and external environment of the patient forms a complete unit. Psychological and social factors are closely related to the occurrence, development, and transformation of diseases [104]. Therefore, evaluation studies in the field of health should not rely solely on descriptive or simple comparative analyses. The broader application of mixed methods to effect evaluation should be considered. Future research on the effects of FDCS should consider synthesising and incorporating multiple evaluation indicators.

Our findings have important implications. This study confirms the positive role of FDCS in Chinese primary care through a systematic synthesis of existing evidence and highlights key factors influencing its sustainable development. However, the evidence is limited by the lack of high-quality, long-term, and interventional studies on the effectiveness of FDCS. Some studies have also reported conflicting findings on the impact on healthcare costs and the quality of primary care. Future research should focus on long-term studies and interventional designs to enable more accurate assessments of the effectiveness of FDCS in managing chronic disease. The government should address the factors affecting the effectiveness of FDCS by optimising incentive mechanisms, improving referral efficiency, increasing the number and quality of FDCS service personnel, and expanding chronic disease service packages. These measure will enhance the comprehensive and sustainable role of FDCS in primary care.

## Limitations and strengths

This review adopted a comprehensive search strategy, retrieved studies from multiple databases, and incorporated all relevant terms. The quality of the included studies was assessed using established tools. However, this review has several limitations. More than half (53.1%) of the included studies focused on the eastern region of China. Although this reflects the policy advantages of the leading pilot reform in primary healthcare, it also highlights the regional limitations of the research samples. The developed economy and dense medical resources in the eastern region have led to significant differences in the implementation of FDCS between the eastern region and the central and western regions. These differences may limit the generalisability of research findings to the entire country, especially to areas with limited medical resources. Research investment in the central and western regions should be increased to facilitate the analysis of the correlations between economic levels, policy support intensity, and FDCS effects and to provide a basis for the formulation of differentiated policies. Second, 71.9% of quantitative studies had medium risks of bias. The short follow-up duration of the cohort studies (mostly 1–2 years) and the lack of intervention designs such as randomised controlled trials also limit the persuasiveness of the evidence. Third, significant differences exist in the measurement tools used for the same concepts (such as QoL and quality of primary care) across the studies. For example, the SF-36, Generic Quality of Life Inventory 74, QoL-BREF, and EQ-5D-3L were used for QoL assessments, and they have different dimensional weights and scoring methods. The differences in the application scenarios of tools such as PCAT and ASPC for quality of primary care assessments have exacerbated the heterogeneity of the research. The consequences of this fragmentation are far-reaching. Methodologically, it undermines the robustness of systematic reviews and meta-analyses, as researchers are forced to either exclude studies with non-conforming tools or pool incomparable data. Practically, it weakens the evidence base for policy-making: decision-makers cannot confidently determine whether observed differences in “QoL improvement” or “quality of primary care” reflect true intervention effects or merely measurement artifacts. Moreover, this inconsistency wastes research resources, as redundant studies are conducted to validate outcomes using overlapping yet incompatible tools, diverting attention from addressing unmet needs in tool development. This review may be affected by publication bias because studies with positive outcomes are more likely to be published. Furthermore, meta-analysis was not performed on the data from the quantitative studies because of the high heterogeneity of the data.

## Conclusions

This review demonstrates that FDCS plays a predominantly positive role in the management of chronic diseases. However, evaluation studies on the long-term effects of FDCS are lacking. Future policy reforms should also address issues such as low doctor motivation, resident concerns, low referral efficiency, and lack of specialised services.

## Supplementary Information

Supplementary Material 1

## Data Availability

The authors confirm that the data supporting the findings of this study are available within the article and its supplementary materials.

## Abbreviations

Abbreviation: Definition
FDCS: Family Doctor Contract Services
QoL: Quality of Life
PCAT: Primary Care Assessment Tool
ASPC: Assessment Survey of Primary Care
HPV: Human Papilloma Virus
