# Supplementary material for: Effectiveness of family doctor contract services for chronic diseases management in China: a mixed-methods systematic review
Source: BMC Prim Care. 2025 Oct 31;26:333. doi: 10.1186/s12875-025-03009-3 (PMC12577023; doi:10.1186/s12875-025-03009-3)
Supplement: Supplementary file 1 — Supplementary Material 1 [file 12875_2025_3009_MOESM1_ESM.docx]

**Appendix**

**Table S1.** Search strategy and databases

| **Database** | **Search strategy** |
| --- | --- |
| PubMed | 1. ("family doctor*") OR ("family physician*") OR ("general practi*")OR（general practice [MeSH]）  2.China  3. (chronic disease*) OR (chronic care) OR (diabetes) OR (hypertension)OR (Chronic Disease[MeSH]) OR (Long-Term Care [MeSH])  4. (English [Language]) OR (Chinese [Language])  5. 1 AND 2 AND 3 AND 4 |
| Web of Science | 1. TS= ("family doctor*") OR ("family physician*") OR ("general practi*") OR（general practice）  2. China  3. TS= (chronic disease*) OR (chronic care) OR (diabetes) OR (hypertension)OR (Chronic Disease) OR (Long-Term Care)  4. 1 AND 2 AND 3 |
| Embase | （'family doctor*' OR 'family physician*' OR 'general practi*'）and （'chronic disease*' OR 'chroniccare' OR 'diabetes' OR 'hypertension' OR 'long-term care'）AND China AND [english]/lim |
| CNKI | (family doctor + family doctor contract + contracted family doctor + family doctor services + family doctor contracted services) AND (chronic disease + [chronic disease prevention and contro](https://context.reverso.net/%E7%BF%BB%E8%AF%91/%E8%8B%B1%E8%AF%AD-%E4%B8%AD%E6%96%87/chronic+disease+prevention+and+control)l + chronic disease management) |
| Wanfang data | (family doctor or family doctor contract or contracted family doctor or Family doctor services or family doctor contracted services） and (chronic disease or [chronic disease prevention and contro](https://context.reverso.net/%E7%BF%BB%E8%AF%91/%E8%8B%B1%E8%AF%AD-%E4%B8%AD%E6%96%87/chronic+disease+prevention+and+control)l or chronic disease management) |

Figure S1 presents regional distribution of the included studies. In China’s regional development strategy, the national division into eastern, middle, and western areas is a comprehensive categorization based on geographic location, level of economic development, and policy orientation. The eastern area includes coastal and more economically developed provinces, the middle area comprises inland provinces, and the western area consists mainly of frontier and less developed regions. Among the 64 studies, two (3.1%) conducted sampling in all three areas, while 17 (26.6%) did not report the study location. Of the remaining 45 studies, 34 (53.1%) were conducted in the eastern area, seven (10.9%) in the middle area, and four (6.3%) in the western area.

**Figure S1.** Regional distributions of the included studies

Figure S2 presents publication trends. All included studies were published after 2013, aligning with the timing of FDCS implementation. Regarding annual publication rates, the highest number of studies was recorded in 2022 (n = 12, 18.7%), followed by 2021 (n = 9, 14.1%) and 2020 (n = 9, 14.1%), which together accounted for approximately 46.9% of all included studies. The marked increase in publications since 2019, and the sustained high output from 2020 to 2022, reflect the growing research interest in FDCS in recent years.

**Figure S2.** Publication trends of included studies

The impact of FDCS on health status and health outcomes was reported in the main text. Tables S2-S9 report the detailed outcomes for each disease area.

Tables S2 and S3 report on the effectiveness of FDCS on hypertension. Among the 26 studies involving hypertension, 11 used the hypertension control rate to evaluate treatment effectiveness, whereas the remaining 14 employed systolic and diastolic blood pressure measurements as evaluation indicators. All included studies reported that, following the signing of a family doctor contract, the evaluation outcomes of contracted residents were superior to those of uncontracted residents. One study assessed awareness of blood pressure measurement through questionnaires administered to 982 patients with hypertension, finding that 85.36% of contracted patients were aware of blood pressure measurement—significantly higher than the proportion observed among uncontracted patients.

**Table S2.** The impact of FDCS on hypertension control rate and awareness

| **Reference** | **Number of uncontracted residents** | **Number of effective residents/ residents with measurement awareness** | **Number of contracted residents** | **Number of effective residents/ residents with measurement awareness** |
| --- | --- | --- | --- | --- |
| **Hypertension control rate** | | | | |
| Sun G, 2021 | 23 | 14 | 25 | 23 |
| Wang J, et al, 2014 | 93 | 53 | 181 | 148 |
| Lin W, et al, 2020 | 179 | 79 | 347 | 191 |
| Li X, 2020 | 104 | 41 | 104 | 67 |
| Zhang Y, et al, 2023 | 107 | 53 | 107 | 68 |
| Li H, 2021 | 273 | 147 | 266 | 183 |
| Wu L, et al, 2020 | 504 | 138 | 1341 | 702 |
| Huang Z, et al, 2015 | 108 | 89 | 126 | 118 |
| Mo H, et al, 2017 | 44 | 31 | 44 | 40 |
| Li Y, et al, 2022 | 53 | 32 | 53 | 48 |
| Shi Z, 2022 | 34 | 21 | 34 | 32 |
| **Blood pressure measurement awareness** | | | | |
| Yan C, et al, 2022 | 702 | 515 | 280 | 239 |

**Table S3.** The impact of FDCS on systolic and diastolic blood pressures measurements

| **Reference** | **Number of uncontracted residents** | **Systolic pressure (mmHg)** | **Diastolic blood pressure (mmHg)** | **Number of contracted residents** | **Systolic pressure (mmHg)** | **Diastolic blood pressure (mmHg)** |
| --- | --- | --- | --- | --- | --- | --- |
| Li H, 2019 | 120 | 135.45±11.27 | 85.46±4.71 | 120 | 124.18±12.35 | 78.52±9.63 |
| Deng L, et al, 2016 | 110 | 142.18±9.75 | 89.59±5.16 | 171 | 135.26±7.49 | 85.34±4.18 |
| Gu Q, 2017 | 55 | 139.37±13.61 | 80.23±10.04 | 55 | 133.48±13.53 | 78.67±7.99 |
| Deng S, 2015 | 200 | 138.31±13.31 | 80.7±10.17 | 200 | 132.5±13.56 | 78.76±8.92 |
| Zhou Z, 2018 | 50 | 138.26±14.72 | 80.11±11.15 | 50 | 132.37±14.64 | 77.55±8.99 |
| Yun Z, 2021 | 32 | 143.54±9.31 | 87.37±5.23 | 32 | 133.49±6.84 | 82.38±4.21 |
| Wei L, et al, 2018 | 100 | 140.1 | 91.57 | 100 | 133.16 | 88.21 |
| Hu Y, et al, 2019 | 150 | 141.6±12.0 | 89.5±8.0 | 150 | 132.0±8.5 | 80.0±6.1 |
| Liu T, et al, 2019 | 219 | 136.83±10.57 | 95.26±9.15 | 252 | 124.86±11.54 | 84.97±10.84 |
| Deng Y, et al, 2022 | 51 | 139.65±16.37 | 88.39±10.94 | 51 | 131.64±12.73 | 80.39±9.87 |
| Jing Y, et al, 2013 | 220 | 138.32±13.32 | 80.71±10.18 | 220 | 132.51±13.57 | 78.77±8.93 |
| Lu X, 2021 | 50 | 135.01±1.11 | 88.90±1.12 | 50 | 127.31±0.92 | 83.34±0.42 |
| Feng J, 2023 | 40 | 142.06±9.51 | 89.67±5.06 | 40 | 135.62±7.43 | 85.22±4.26 |
| Yang Z, 2022 | 51 | 141±9 | 86±7 | 51 | 137±8 | 80±5 |

Tables S4 and S5 report on the effectiveness of FDCS on diabetes. Among the 21 studies involving patients with diabetes, 11 assessed the role of family doctors using the diabetes control rate, six considered fasting blood glucose only, and the remaining four used both fasting blood glucose and 2-hour postprandial glucose as evaluation indicators. All studies reported that, following the implementation of the FDCS policy, contracted residents demonstrated better indicators than uncontracted residents.

**Table S4.** The impact of FDCS on diabetes control rate

| **Reference** | **Number of uncontracted residents** | **Number of effective residents** | **Number of contracted residents** | **Number of effective residents** |
| --- | --- | --- | --- | --- |
| Sun G, 2021 | 25 | 15 | 23 | 20 |
| Wang J, et al, 2014 | 93 | 51 | 181 | 149 |
| Lin W, et al, 2020 | 72 | 55 | 213 | 113 |
| Li X, 2020 | 104 | 37 | 104 | 74 |
| Zhang Y, et al, 2023 | 107 | 95 | 107 | 103 |
| Li H, 2021 | 67 | 26 | 80 | 45 |
| Wu L, et al, 2020 | 195 | 29 | 481 | 181 |
| Huang Z, et al, 2015 | 108 | 87 | 126 | 120 |
| Mo H, et al, 2017 | 44 | 29 | 44 | 39 |
| Li Y, et al, 2022 | 53 | 35 | 53 | 49 |
| Shi Z, 2022 | 34 | 20 | 34 | 30 |

**Table S5.** The impact of FDCS on fasting blood glucose and 2-h postprandial glucose

| **Reference** | **Number of uncontracted residents** | **Fasting blood glucose (mmol/L)** | **2-h postprandial glucose (mmol/L)** | **Number of contracted residents** | **Fasting blood glucose (mmol/L)** | **2-h postprandial glucose (mmol/L)** |
| --- | --- | --- | --- | --- | --- | --- |
| Zhu X, 2020 | 200 | 10±1.5 | - | 200 | 7.2±1.6 | - |
| Li H, 2019 | 120 | 6.73±0.58 | 9.65±1.24 | 120 | 5.14±0.45 | 7.96±1.09 |
| Deng L, et al, 2016 | 110 | 7.16±1.04 | 9.82±2.06 | 171 | 6.15±0.86 | 8.04±1.28 |
| Gu Q, 2017 | 55 | 7.65±2.22 | - | 55 | 7.24±1.51 | - |
| Deng S, 2015 | 200 | 7.62±2.24 | - | 200 | 7.22±1.48 | - |
| Zhou Z, 2018 | 50 | 7.64±2.33 | - | 50 | 7.13±1.62 | - |
| Yun Z, 2021 | 32 | 7.19±0.31 | 9.89±1.04 | 32 | 6.24±0.42 | 8.01±1.04 |
| Deng Y, et al, 2022 | 51 | 7.15±0.84 | 9.61±1.41 | 51 | 6.14±0.43 | 8.04±1.12 |
| Jing Y, et al, 2013 | 220 | 7.63±2.25 | - | 220 | 7.23±1.49 | - |
| Xu C., et al, 2022 | 1155 | 6.8±1.0 | - | 1155 | 6.6±0.9 | - |

Table S6 presents the effectiveness of FDCS on lipid control. Four studies assessed lipid levels, and overall, they demonstrated improvements in lipid profiles among contracted residents. Three of these studies (75%) reported lipid control rates; pooled data from these studies indicated a higher control rate in contracted patients than in uncontracted patients (74.69% vs 48.98%). The remaining study (25%) reported four biochemical markers and found that contracted patients had more favourable levels of total cholesterol (3.20 vs 5.08 mmol/L), triglycerides (1.39 vs 1.88 mmol/L), low-density lipoprotein cholesterol (1.53 vs 2.42 mmol/L), and high-density lipoprotein cholesterol (1.40 vs 1.06 mmol/L) compared with uncontracted patients.

**Table S6.** The impact of FDCS on lipid control rate

| **Reference** | **Number of uncontracted residents** | **Number of effective residents** | **Number of contracted residents** | **Number of effective residents** |
| --- | --- | --- | --- | --- |
| Li X, 2020 | 104 | 37 | 104 | 74 |
| Zhang Y, et al, 2023 | 107 | 63 | 107 | 78 |
| Shi Z, 2022 | 34 | 20 | 34 | 31 |

Eleven studies reported results related to quality of life (QoL) and the detailed results are presented in Tables S7-S9. Five instruments were used across these studies: the Short Form 36 (SF-36), EQ-5D-3L, Generic Quality of Life Inventory 74, Diabetes-Specific Quality of Life Scale, and QOL-BREF. Analysis of questionnaire scores indicated that contracted residents had better QoL scores than uncontracted residents.

In the assessment using the Diabetes-Specific Quality of Life Scale, a higher score indicates a lower QoL. The scores of 200 uncontracted residents in physical function (24.8±6.1 vs 19.6±5.2), psychological function (22.1±4.7 vs 18.1±4.6), social relations (5.9±2.3 vs 4.9±1.1), treatment satisfaction (6.1±1.9 vs 5.0±1.1), and total scores (59.1±7.9 vs 50.1±6.0) were all higher than those of the 200 contracted residents. In the assessment using the QOL-BREF, a higher score indicates a better QoL. The scores of 44 uncontracted residents in physical function (10.08±1.54 vs 11.98±1.75), psychological function (10.87±1.76 vs 12.43±2.12), social function (10.45±1.87 vs 13.21±2.43), and environmental (9.12±1.87 vs 10.98±2.02) were all lower than those of the 44 contracted residents. The specific results of other studies were detailed in the table.

**Table S7.** The impact of FDCS on QoL (SF-36)

| **Reference** | Whang L, et al, 2022 | Chen Y, 2020 | Han Y, 2021 | Liu T, et al, 2019 | Li Y, et al, 2022 |
| --- | --- | --- | --- | --- | --- |
| **Number of uncontracted residents** | 382 | 40 | 40 | 219 | 53 |
| **Physical functioning** | 90.92 | - | 90.45±2.45 | - | 72.65±6.45 |
| **Role-physical** | 88.86 | - | 91.56±2.43 | - | - |
| **Bodily pain** | 64.99 | - | - | - | - |
| **General health** | 43.71 | - | - | - | - |
| **Vitality** | 57.34 | - | - | - | - |
| **Social functioning** | 20.00 | - | - | - | 73.25±6.25 |
| **Role-emotional** | 51.04 | - | 91.62±2.34 | - | - |
| **Mental health** | 50.96 | - | 90.18±2.86 | - | 75.65±5.21 |
| **Total scores** | - | 70.17±3.21 | - | 65.14±7.99 | - |
| **Number of contracted residents** | 382 | 40 | 40 | 252 | 53 |
| **Physical functioning** | 87.58 | - | 92.23±2.11 | - | 86.58±5.26 |
| **Role-physical** | 85.67 | - | 93.43±2.13 | - | - |
| **Bodily pain** | 68.51 | - | - | - | - |
| **General health** | 42.87 | - | - | - | - |
| **Vitality** | 51.37 | - | - | - | - |
| **Social functioning** | 17.18 | - | - | - | 90.25±5.21 |
| **Role-emotional** | 61.63 | - | 93.46±2.13 | - | - |
| **Mental health** | 50.76 | - | 92.13±2.46 | - | 89.62±4.25 |
| **Total scores** | - | 84.23±2.34 | - | 81.24±6.87 | - |

**Table S8.** The impact of FDCS on QoL (EQ-5D-3L)

| **Reference** | Lai S, et al, 2021 | Li Z, et al, 2021 |
| --- | --- | --- |
| **Number of uncontracted residents** | 2182 | 855 |
| **Mobility** | 0.1498 | 0.281 |
| **Selfcare** | 0.0549 | 0.139 |
| **Activity** | 0.0755 | 0.276 |
| **Pain** | 0.2707 | 0.547 |
| **Anxiety** | 0.1196 | 0.243 |
| **EQ-5D values** | 0.8995 | 0.831 ± 0.181 |
| **Number of contracted residents** | 2430 | 355 |
| **Mobility** | 0.0947 | 0.172 |
| **Selfcare** | 0.0383 | 0.070 |
| **Activity** | 0.0556 | 0.169 |
| **Pain** | 0.2008 | 0.473 |
| **Anxiety** | 0.0897 | 0.175 |
| **EQ-5D values** | 0.9355 | 0.875 ± 0.141 |

Note: The data in the table represent the frequency of “moderate problem” in EQ-5D-3L dimensions, and studies employed the Chinese time trade-off values for EQ-5D-3L to measure the utility values of the EQ-5D-3L

**Table S9.** The impact of FDCS on QoL (Generic Quality of Life Inventory 74)

| **Reference** | Chen J, 2018 | Chan W, et al, 2023 |
| --- | --- | --- |
| **Number of uncontracted residents** | 100 | 48 |
| **Physical function** | 51.9±4.7 | 72.97±11.03 |
| **Psychological function** | 38.6±5.2 | 73.08±11.00 |
| **Social function** | 52.8±4.9 | 72.20±10.65 |
| **Physical life** | 58.6±3.7 | 71.55±9.86 |
| **Number of contracted residents** | 100 | 48 |
| **Physical function** | 63.2±3.1 | 83.06±10.52 |
| **Psychological function** | 45.9±4.2 | 82.96±10.24 |
| **Social function** | 61.7±4.8 | 81.98±10.33 |
| **Physical life** | 52.1±4.5 | 81.59±10.21 |
